# PKC/ROS-Mediated NLRP3 Inflammasome Activation Is Attenuated by *Leishmania* Zinc-Metalloprotease during Infection

**DOI:** 10.1371/journal.pntd.0003868

**Published:** 2015-06-26

**Authors:** Marina Tiemi Shio, Jan Gregor Christian, Jee Yong Jung, Kwang-Poo Chang, Martin Olivier

**Affiliations:** 1 Department of Microbiology and Immunology, McGill University, Montréal, Québec, Canada; 2 Department of Microbiology, Immunology and Parasitology, Universidade Federal de São Paulo, São Paulo, Brazil; 3 McGill International Tuberculosis (TB) Centre and the Research Institute of the McGill University Health Centre, Montréal, Québec, Canada; 4 Chicago Medical School, Rosalind Franklin University of Medicine and Science, North Chicago, Illinois, United States of America; Harvard School of Public Health, UNITED STATES

## Abstract

Parasites of the *Leishmania* genus infect and survive within macrophages by inhibiting several microbicidal molecules, such as nitric oxide and pro-inflammatory cytokines. In this context, various species of *Leishmania* have been reported to inhibit or reduce the production of IL-1β both *in vitro* and *in vivo*. However, the mechanism whereby *Leishmania* parasites are able to affect IL-1β production and secretion by macrophages is still not fully understood. Dependent on the stimulus at hand, the maturation of IL-1β is facilitated by different inflammasome complexes. The NLRP3 inflammasome has been shown to be of pivotal importance in the detection of danger molecules such as inorganic crystals like asbestos, silica and malarial hemozoin, (HZ) as well as infectious agents. In the present work, we investigated whether *Leishmania* parasites modulate NLRP3 inflammasome activation. Using PMA-differentiated THP-1 cells, we demonstrate that *Leishmania* infection effectively inhibits macrophage IL-1β production upon stimulation. In this context, the expression and activity of the metalloprotease GP63 - a critical virulence factor expressed by all infectious *Leishmania* species - is a prerequisite for a *Leishmania*-mediated reduction of IL-1β secretion. Accordingly, *L*. *mexicana*, purified GP63 and GP63-containing exosomes, caused the inhibition of macrophage IL-1β production. *Leishmania*-dependent suppression of IL-1β secretion is accompanied by an inhibition of reactive oxygen species (ROS) production that has previously been shown to be associated with NLRP3 inflammasome activation. The observed loss of ROS production was due to an impaired PKC-mediated protein phosphorylation. Furthermore, ROS-independent inflammasome activation was inhibited, possibly due to an observed GP63-dependent cleavage of inflammasome and inflammasome-related proteins. Collectively for the first time, we herein provide evidence that the protozoan parasite *Leishmania*, through its surface metalloprotease GP63, can significantly inhibit NLRP3 inflammasome function and IL-1β production.

## Author Summary

*Leishmania* parasites are the causative agent of leishmaniasis, a wide spread disease in tropical and subtropical areas. The microorganisms have been shown to be well-adapted to their hosts and are able to enter their target cells where they replicate themselves. To ensure these processes, *Leishmania* disrupts a multitude of cellular signals and protective mechanisms, which overall attenuates immune responses against the parasites. A key factor for inflammatory processes, also during infections, is IL-1β. As previous studies suggested a dysregulation of IL-1β levels after infection with *Leishmania* parasites, we herein investigated the underlying mechanisms. Our work reveals that *Leishmania* suppressing IL-1β production through its virulence factor GP63. Furthermore, our data suggests that the parasites can dampen the maturation of IL-1β after different stimuli. In this regard we established a role for the suppression of the kinase PKC and the generation of reactive oxygen species, as well as the cleavage of cellular proteins that are important for IL-1β-generation. Thus, we here present a novel aspect for how *Leishmania* parasites can counteract host protective mechanisms.

## Introduction

*Leishmania* parasites, which are the causative agent of leishmaniasis, are able to both survive and proliferate within macrophages. The protozoan parasites evolved strategies to avoid phagocyte activation during infection by seizing control of key signaling pathways [1,2]. Studies previously implicated the metalloprotease GP63—a major virulence factor of *Leishmania* parasites—in a variety of parasite survival mechanisms. In this context, GP63 has been suggested to affect amongst others *Leishmania* binding to macrophages, phagocytosis of parasites, evasion of complement-mediated lysis and protozoan migration through the extracellular matrix [1,3]. Furthermore, GP63 has been identified as a key *Leishmania* virulence factor that modulates cellular signalling through the subversion of host protein tyrosine phosphatase (PTP) function [4,5,6]. In this context GP63-mediated PTP-cleavage, results in the activation of the respective phosphatases. This mechanism was identified for the SH2 domains-containing protein tyrosine phosphatase (SHP-1) and protein-tyrosine phosphatase 1B (PTP-1B) [6]. Besides phosphatases, GP63 has been shown to cleave other targets within the cells including kinases like TAB 1 and transcription factors, including AP-1 and NF-κB [7,8]. The importance of the host PTP-modulation and the subsequent inhibition of signaling pathways is emphasized by the observation that key pro-inflammatory mediators such as nitric oxide (NO), IL-6 and TNFα were subsequently downregulated by *Leishmania* [4,5,9].

Another factor of pivotal importance for inflammatory processes that has been studied in the past in the context of *Leishmania* infections is IL-1β. In this regard *Leishmania* infections have been reported in a variety of studies to alter IL-1β production dependent on the parasite species used [4,10,11,12,13]. This included the deregulation of the IL-1β release due to parasite infections upon usage of known IL-1β inducers like LPS, IFN-γ or nigericin. However, the means used by the parasites to interfere with inflammasome activation remain unclear to date.

IL-1β is translated as an inactive precursor—pro-IL-1β (31 kDa), which is processed into active IL-1β by the multi-protein inflammasome-complex upon stimulation of the cells. Integral components of the inflammasome complexes are caspase-1, responsible for proteolytic cleavage of the IL-1β precursor, [14] a member of the NOD-like receptor (NLR) family, which acts as the sensor component of the inflammasome and ASC, a CARD/PYD protein that serves as a docking and activation platform for caspase-1 and the respective NLR [14]. Dependent on the NLR-protein within the complex, inflammasomes have been shown to respond to a variety of stimuli including bacterial and viral pathogen associated molecular patterns (PAMPs) like microbial nucleic acids or proteins and danger associated molecular patterns (DAMPs) [15,16]. In context of the latter, the NLRP3-containing inflammasome was found to be critical for the recognition of inorganic crystals such as malarial hemozoin (HZ), silica and asbestos, as well as other DAMPs like cardiolipin, ATP and uric acid (MSU) [17,18,19,20]. Canonical NLRP3-inflammasome activation requires two signals. The first signal results in an increased expression of pro-IL-1β and the NLR protein as basal levels are insufficient to facilitate inflammasome activation. Typical initial signals are relayed through pattern recognition receptors such as TLRs or receptors for cytokines (e.g. type I IFNs) [15]. Furthermore, inducers of PKC and MAPK-dependent signaling such as phorbol myristate acetate (PMA) have been used as first signals for inflammasome activation *in vitro* [21,22]. The second signal induces the oligomerisation and complex formation of the inflammasome that allows the processing of pro-IL-1β. Complex formation and activation can be triggered in different ways including ROS generation, potassium efflux, lysosomal damage and mitochondrial destabilization or damage [23]. Following this rationale of NLRP3 inflammasome organization and activation, modulation—for instance after *Leishmania* infections—may take place at the level of priming, complex assembly or complex activation as has been shown for a variety of other pathogens that interfere with inflammasomes.

In this study we report that *Leishmania* parasites, through its virulence factor GP63 inhibit IL-1β production and secretion induced by different NLRP3 inflammasome activators. Importantly, this was observable in both a murine and human model system for Leishmania infection. Dysregulation is achieved due to GP63-dependent interference with signaling pathways upstream of the inflammasome, which affect ROS generation. In this context our data suggests PKC signaling and its downregulation is pivotal for the *Leishmania*-mediated downregulation of inflammasome activation. In addition, *Leishmania* GP63 also seems to specifically target components of the inflammasome for proteolytic cleavage that is most likely the basis for the suppression of IL-1β production after ROS-independent activation of the NLRP3 inflammasome.

## Materials and Methods

### Cell lines and reagents

L929 and THP-1 cell lines were obtained from ATCC (Manassas, VA, USA). Cells were cultured in RPMI-1640 medium supplemented with Penicillin-Streptomycin-Glutamine (PSG; both reagents from Wisent, Saint-Jean Baptiste, QC, Canada) and fetal bovine serum (FBS; Gibco Burlington, ON, Canada). In some experiment the culture medium was exchanged to MEM Alpha medium (Gibco, Burlington, ON, Canada). Reagents used included linezolid, ATP, silica and hemin (> 99% of purity; Sigma-Aldrich, Oakville, ON, Canada); GÖ6983 (Biomol, Farmingdale, NY, USA); asbestos (Structure Probe, West Chester, PA, USA), MSU (Alexis Biochemical, Farmingdale, NY, USA); protease inhibitor cocktail (Roche, Mississauga, ON, Canada) and PVDF membrane (BIO-RAD, Mississauga, ON, Canada). All others unlisted or not indicated reagents were purchased from Sigma-Aldrich (Oakville, ON, Canada) Antibodies used in experiments included anti-human NLRP3 and ASC (Alexis Biochemical, Farmingdale, NY, USA), anti-human pro-IL-1β, anti-human and murine caspase-1 (Santa Cruz; Dallas, TX, USA), anti-phospho-tyrosine/HRP (eBiosciences; San Diego, CA, USA), anti-human mature IL-1β (Cell signaling Technology, Danvers, MA, USA; Rockland-Immunochemicals, Limerick, PA, USA), phospho(Ser)-PKC-substrate antibody (Cell signaling Technology; Danvers, MA, USA), anti-GP63 (obtained from Dr. McMaster, University of British Columbia, Vancouver, Canada) and anti-murine IL-1β (R&D systems, Minneapolis, MN).

### Synthetic HZ production

Synthetic HZ was generated as previously described [24,25]. Briefly, 0.8 mmol crystalline hemin (>99% of purity) was dissolved in degassed NaOH (0.1M) for 30 minutes with stirring. After, the pH was adjusted with propionic acid to 4 and the material was allowed to anneal at 70°C for 18 hrs. The supernatant was removed and the crystals were incubated three times with NaHCO_3_ (0.1M) for three hours. In between incubations, samples were briefly washed with milliQ H_2_O. Thereafter, the crystals were washed three times with methanol and milliQ H_2_O in an alternating fashion. Subsequently, the samples were dried in a vacuum oven overnight over phosphorous pentoxide (Sigma-Aldrich, Oakville, ON, Canada). Synthetic HZ samples were analyzed by X-ray powder diffraction, scanning electron microscopy (SEM), and infra-red spectroscopy to characterize the crystalline state of HZ.

### *Leishmania* culture and *Leishmania*-conditioned medium

*Leishmania major* (*L*. *major*), *L*. *mexicana*, *L*. *major* GP63 knock out (GP63 KO), and *L*. *major* GP63 rescue (the GP63 gene was inserted into *L*. *major* GP63 KO parasites [26]) were used in different experimental setups. All *Leishmania* parasites were maintained at 25°C in SDM-79 culture medium supplemented with 10% FBS by bi-weekly passage and used for different applications after 6–7 days of culture (stationary phase). Stationary phase parasites were either used to infect macrophages (at a ratio of 20:1), to recover the culture supernatant for GP63 (*L*. *mexicana*) purification or to generate parasite secretome or exosome preparations. To generate *Leishmania*-conditioned medium (LCM), all species of *Leishmania* were adapted to grow in DMEM medium (Wisent, Saint-Jean Baptiste, QC, Canada) supplemented with 10% of FBS and 1% of PSG. After 7 days in culture, LCM was collected by centrifugation of parasite cultures (1,000 *x g*, 5 min) and subsequent filtration with 0.22 μm filters.

### GP63 purification

*Leishmania* GP63 was purified using an immunoaffinity column. The antibodies used to purify *Leishmania* GP63 were specific to *L*. *mexicana* GP63 [27]. The antibody was cross-linked using the Affi-Gel HZ Immunoaffinity kit (BIO-RAD, Mississauga, ON, Canada). GP63 was purified from the supernatant of stationary *L*. *mexicana* cultures and concentrated by centrifugation using Amicon Ultra centrifugational filters (EMD Millipore, Etobicoke, ON, Canada) at 4,000 rpm for 10 minutes and stored at -80°C.

### Exoproteome and exosome preparation

Exoproteome was prepared as described previously [28]. Briefly, stationary *L*. *mexicana* promastigotes were washed 3 times with PBS, incubated in phenol red-free and serum free DMEM (Wisent, Saint-Jean Baptiste, QC, Canada) for 4 hrs and culture supernatants were centrifuged twice at 4,000 rpm for 10 min. Subsequently, the material was either concentrated using 10 kDa cut off Amicon Ultra centrifugational filters (EMD Millipore, Etobicoke, ON, Canada) and used as secretome preparations or centrifuged (100,000 *x g*, 60 min at 4°C) to isolate exosomes. Protein concentration was determined using Bradford reagent (BIO-RAD, Mississauga, ON, Canada).

### THP-1 culture and stimulation

THP-1 cells were cultured with RPMI-1640 medium supplemented with 10% FBS, 1% PSG, 50 μM of 2-β-mercaptoethanol, 4.5 g/L of Glucose and 1 mM sodium pyruvate. For THP-1 differentiation 1.5 x10^6^ cells/mL were incubated with 0.5 μM of PMA. After three hrs cells were washed, plated (0.75 x 10^6^ cells/mL in 6 wells plates) and incubated for 20–24 hrs. As a consequence the phagocytic properties of the cells were increased and expression of inflammasome proteins and pro-IL-1β was induced. In some experiments THP-1 cells were incubated with 50 ng/ml PMA for 24 hrs. Cells were infected with indicated *Leishmania* spp. at a ratio of 1:20 (macrophages:parasites) or incubated with purified GP63, secretome preparations, exosomes preparations or *Leishmania* culture medium (LCM). Cells were washed after times of infection dependent on the experimental setup and the medium was replaced with MEM Alpha medium without FBS. Cells were subsequently stimulated with indicated concentrations of HZ, silica, asbestos, MSU or ATP for 6 hrs. Linezolid was incubated for 18 hrs.

### Culture and stimulation of bone-marrow derived macrophages (BMDM)

Bone marrow cells were obtained by flushing out the femurs and tibias from 6 weeks old C57Bl/6 mice. Subsequently, erythrocytes were lysed using NH_4_Cl (155 mM) in Tris/HCl (10 mM), pH 7.2. Bone marrow derived cells were counted, seeded and incubated in RPMI-1640 medium supplemented with 1% of PSG, 10% FBS and 30% (v/v) L929 cell culture supernatant. Cells were cultured for 7 days, exchanging the culture media every second day. For assays, BMDM were harvested and seeded (0.75 x 10^6^/mL) in RPMI medium supplemented with 5% FBS and 1% of PSG. The following day, cells were primed with LPS (100 ng/ml, 3 hrs) and infected with *Leishmania* spp. at a ratio of 1:20 (macrophages:parasites). After infection for variable time periods dependent on the experimental setup, cells were washed, medium was replaced with MEM-Alpha without FBS and cells were stimulated with indicated concentrations of HZ or linezolid for 6 hrs or 18 hrs respectively.

### Supernatant precipitation, cell extract generation, and immunoblotting

Supernatants were collected at indicated time points and proteins were precipitated with trichloroacetic acid at a final concentration of 10%. Precipitated proteins were dissolved in Tris/HCl 0.1 mM pH 8.0 and laemmli sample buffer [29]. Cell extracts were obtained by lysing cells with either Igepal (Sigma-Aldrich, Oakville, ON, Canada) containing lysis buffer (1% Igepal in PBS, 20% Glycerol, protease inhibitor cocktail, 2 mM Na_3_VO_4_ and 1 mM NaF) or for caspase-1 detection Triton-X-100 (Fisher Scientific, Walham, MA, USA) containing lysis buffer (1% Triton-X-100 in 10 mM Tris/HCl pH 7.5, 150 mM NaCl, 5 mM EDTA and protease inhibitor cocktail). Supernatant and cell lysate samples were subjected to SDS-PAGE and immunoblot analysis. SDS-PAGE/Immunoblot: SDS-PAGE and Immunoblot were performed following protocols previously published [30]. For the detection of caspase-1 p10, 4–12% NuPAGE gels (Invitrogen) were used. After protein transfer onto PVDF membranes, detection of target proteins was achieved through specific primary antibodies and matched secondary HRP-conjugated antibodies.

### ELISA

NUNC maxisorb 96 well plates (Nalge NUNC, Richester NY, USA) were coated with 100 μl/well of capture antibody (SET TO GO kit, eBiosciences, San Diego, CA, USA) overnight and blocked with 200 μl/well assay diluent solution 1 hr at RT. After blocking, 100 μl of standard proteins or samples were added to each well and incubated for 2 hrs. After 5 washes, 100 μl/well of detection antibody were added and incubated 1 hr at RT. For cytokine detection, 100 μl/well of Avidin-HRP were added and incubated for 30 min. Afterwards, 100 μl/well of substrate solution were added for 15 min. 50 μl of stopping solution were added and plates were read at 450 nm in an ELISA reader (Elmer EnSpire Multimode Plate Reader, Perkin Elmer, Waltham, MA, USA) and concentrations were calculated according to a standard curve.

### ROS measurement

PMA-differentiated THP-1 cells (0.1x10^6^ cell/100 μl) were seeded in opaque 96 well plates. Cells were infected as indicated with *Leishmania* parasites for 2 hrs. Cells were washed with PBS and incubated with phenol red free RPMI (Wisent, Saint-Jean Baptiste, QC, Canada) containing 20 mM of 2,7-dichlorofluorescein diacetate—DCFH-DA (Sigma-Aldrich, Oakville, ON, Canada) for 10 min at 37°C. Subsequently, cells were stimulated as indicated adding inflammasome activators. The rate of DCFH-DA oxidation to DCF was observed with a SpectraMax M3 (Molecular Devices, Sunnyvale, CA, USA) fluorescent plate reader at a 488 nm excitation wavelength and a 525 nm emission wavelength.

### Statistical analysis

Unpaired Student’s t-test was used when comparing two groups. The differences were considered significant for p < 0.05. Statistical analysis was performed using Prism 5.00 software (GraphPad, San Diego, CA).

### Ethics statement

C57BL/6 mice were purchased from Charles River Laboratories and Jackson Laboratories, and were kept in pathogen-free housing. All research involving mice was carried out according to the regulations of the Canadian Council of Animal Care and was approved by the McGill University Animal Care Committee under ethics protocol number 4859. Mice were euthanized using CO_2_ asphyxiation followed by cervical dislocation.

## Results

### *Leishmania* inhibits IL-1β production

**Fig 1:**
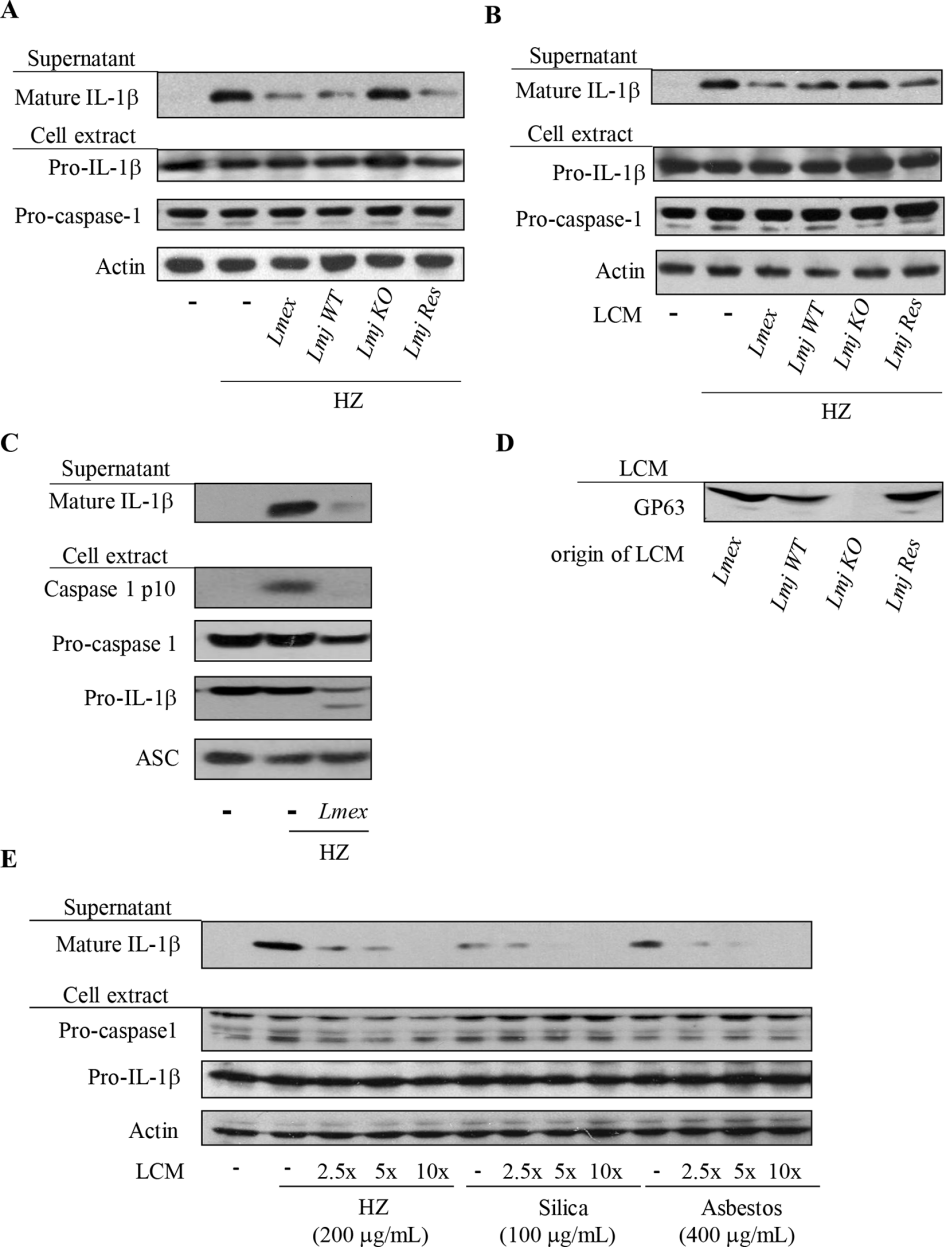
*Leishmania* parasites inhibit IL-1β production induced by HZ in GP63-dependent manner. PMA-differentiated THP-1 cells (1x10^6^ cells/mL) were pre-infected with *Leishmania* parasites (**A**) or incubated (**B**) with culture medium (LCM) from 7 days old parasite cultures of either *Leishmania mexicana* (*Lmex*), *L*. *major* GP63 wild type (*Lmj WT*), *L*. *major* GP63^-/-^ (*Lmj KO*) or *L*. *major* GP63 rescue (*Lmj Res*). Afterwards, cells were washed and stimulated with 200 μg/mL of HZ. (**C**) Bone-marrow derived macrophages (1.5x10^6^ cells/mL) were pre-infected for 2 hrs with *Leishmania mexicana* (*Lmex*). Following infection, cells were washed and stimulated with 200 μg/mL of HZ. After 6 hrs of incubation, supernatant and cell extracts were collected and subjected to Western blot analysis with the indicated antibodies. Data shown is representative of two independent experiments. (**D**) Western blot of *Leishmania* culture medium (LCM) for GP63. Data shown is representative of two independent experiments. (E) PMA-differentiated THP-1 cells (1x10^6^ cells/mL) were incubated with different concentrations of LCM from 7 days old parasite cultures of *L*. *mex*icana. Afterwards, cells were washed and stimulated with either 200 μg/mL of HZ, 200 μg/mL of silica or 200 μg/mL of asbestos for 6 hrs. Supernatants and cell extracts were collected and subjected to Western blot analysis with the indicated antibodies.

Upon activation macrophages can produce a large array of pro-inflammatory molecules including IL-1β, which is produced by inflammasome complexes. The NLRP3 inflammasome acts as an intracellular signaling platform which is able to sense a variety of exogenous signals like asbestos, silica [17] as well as the malarial pigment HZ [20,31] and DAMPs such as ATP [18] and MSU [21]. In initial dose-response experiments using synthetic HZ we confirmed the ability of HZ to induce IL-1β maturation in PMA-differentiated THP-1 cells (S1 Fig). The observed, dose-dependent IL-1β secretion was comparable to the results obtained in the case of silica or MSU treatment of cells. Infection of THP-1 cells with *Leishmania* did not induce IL-1β release as shown for *L*. *mexicana* (S2 Fig). *Leishmania* parasites are well known for their ability to block and inhibit various microbicidal functions of macrophages [32], therefore we sought to elucidate whether infections with *Leishmania* would inhibit IL-1β production by macrophages, which has been indicated to possibly serve a host protective role in murine models of infection [33,34]. As previously introduced the NLRP3 inflammasome activator HZ causes the IL-1β maturation and secretion in PMA-differentiated macrophages. Pre-infection with *L*. *mexicana* and *L*. *major*, revealed a parasite-dependent block of IL-1β maturation and release (Fig 1A). The impaired production of IL-1β was not restricted to a system of human cell culture but was also observable when parasite infection preceded inflammasome activation in murine BMDMs (Fig 1C). In those experiments due to the availability of antibodies the processing of caspase-1 into its active fragments p10 and p20 could be observed. Notably processing of caspase-1 was absent after infection of cells possibly due to the lack of inflammasome activation or complex formation. Identical experimental setups for infections as in Fig 1A using *L*. *major GP63* KO *and L*. *major GP63 rescue* parasites support the hypothesis that *Leishmania*’s capability to inhibit IL-1β maturation and release was GP63-dependent (Fig 1A–1E). Thus, IL-1β secretion was not impaired in the absence of the protease (Fig 1A). This finding was further supported by experiments using pretreatment of THP-1 cells with *Leishmania* culture supernatant (LCM) instead of parasites (Fig 1B). The attenuated IL-1β secretion coincided with the presence of GP63 in the LCM. We were able to show that LCM of *L*. *mexicana* and *L*. *major* GP63 wild type parasite cultures contained GP63 (Fig 1D). To further evaluate the impact of secreted leishmanial factors like GP63 on IL-1β production the supernatant of *L*. *mexicana* cultures was concentrated and used in titration experiment on PMA-differentiated THP-1 cells (Fig 1E). IL-1β maturation and release were stimulated by a variety of known NLRP3 inflammasome inducers, namely HZ, silica and asbestos. In all cases we observed an inhibition of IL-1β secretion in a dose-dependent manner by the *L*. *mexicana* culture supernatant. *Leishmania* GP63 can be found either intracellular in the protozoan endoplasmic reticulum, membrane bound via a GPI anchor or secreted without the GPI anchor. During infection GPI anchored, membrane bound GP63 (GPI-GP63) can also be cleaved and released into the supernatant [2,35]. Therefore, the similarities of our results using either *Leishmania* infections or *Leishmania* culture supernatant are most likely to be attributed to the presence and activity of GP63.

**Fig 2:**
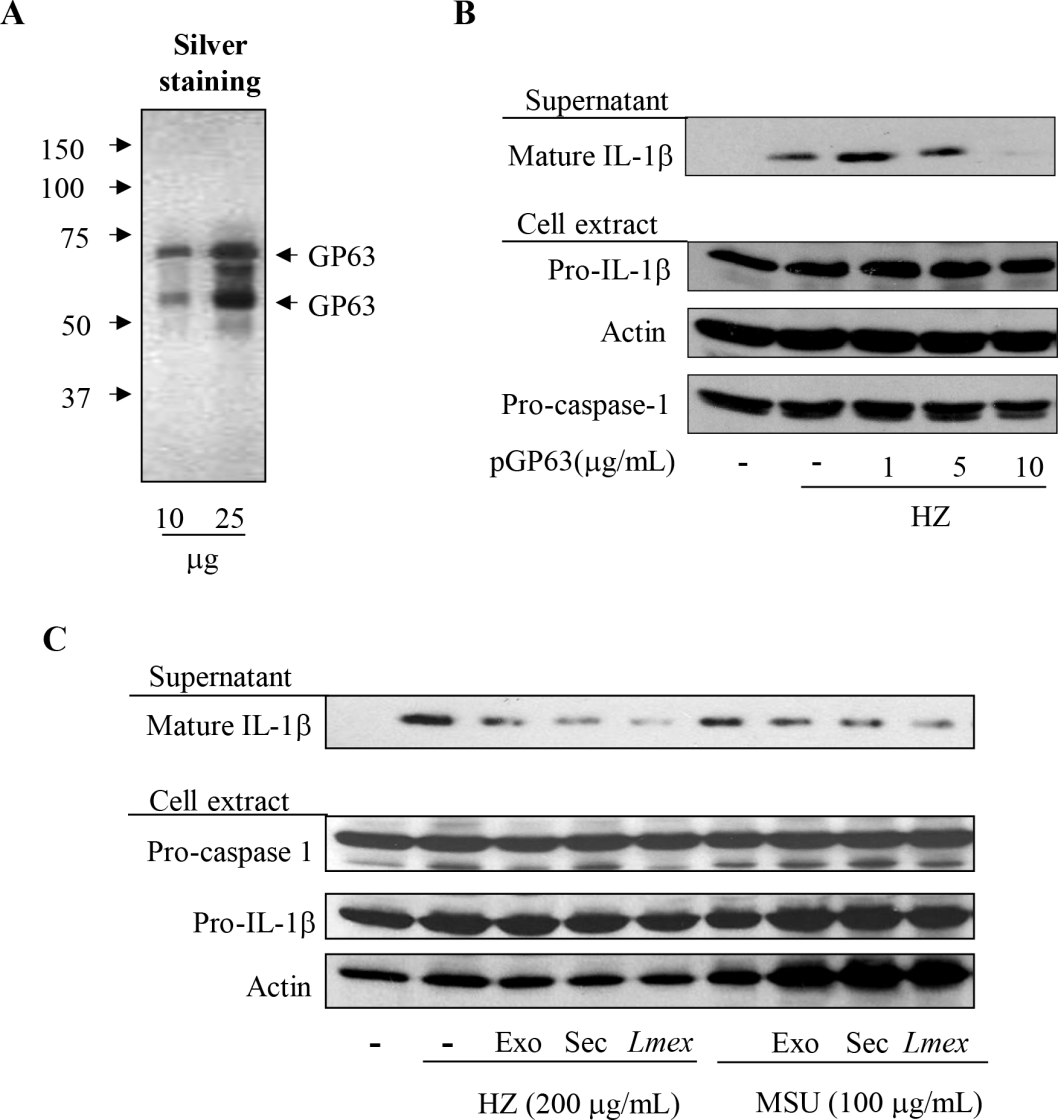
Purified GP63 and leishmanial exosomes containing GP63 inhibit IL-1β maturation induced by inflammasome activators. Silver staining of purified GP63 (**A**). PMA-differentiated THP-1 cells (1x10^6^ cells/mL) were pre-treated (2 hrs) with or without the indicated concentration of purified GP63 (pGP63) (**B**) or either leishmanial exosome (exo) or secretome (sec) (**C**) for 2 hrs and stimulated with 200 μg/mL of the malarial pigment—HZ, or MSU as specified. After 6 hrs of incubation, supernatant and cell extracts were collected and subjected to Western blot analysis with the indicated antibodies. Data shown is representative of three independent experiments. Materials containing GP63 leads to inhibition of inflammasome activation.

In recent years, several studies have described the observation that proteins are secreted as exosecretome by *Leishmania* parasites upon 37°C temperature shock [28] or as exosomes during the culture of parasites [36,37]. Both *Leishmania* secretome and exosomes have been shown to contain GP63. Thus, we evaluated whether leishmanial secretome or exosome preparations would inhibit IL-1β maturation and secretion. As expected the results were in accordance with the data acquired using parasite infections and culture supernatant treatment of cells, with both secretome and exosomes inhibiting IL-1β production induced by either HZ or MSU (Fig 2C). The importance of the metalloprotease GP63 was clearly demonstrated through experiments using purified GPI-GP63 (Fig 2A and 2B) from *L*. *mexicana* stationary phase cultures to pretreat THP-1 cells. This resulted in an attenuation of the HZ-induced IL-1β maturation and its release (Fig 2B) identical to infection experiments previously shown. Collectively, these results provide convincing evidence that *Leishmania* GP63 is the causative factor for an impaired IL-1β production by the NLRP3 inflammasome complex, which was observed after infections with *Leishmania* parasites prior to cell stimulation.

### PKC-dependent signaling links HZ-dependent ROS-generation and inflammasome activation but is impaired by *Leishmania* infections

**Fig 3:**
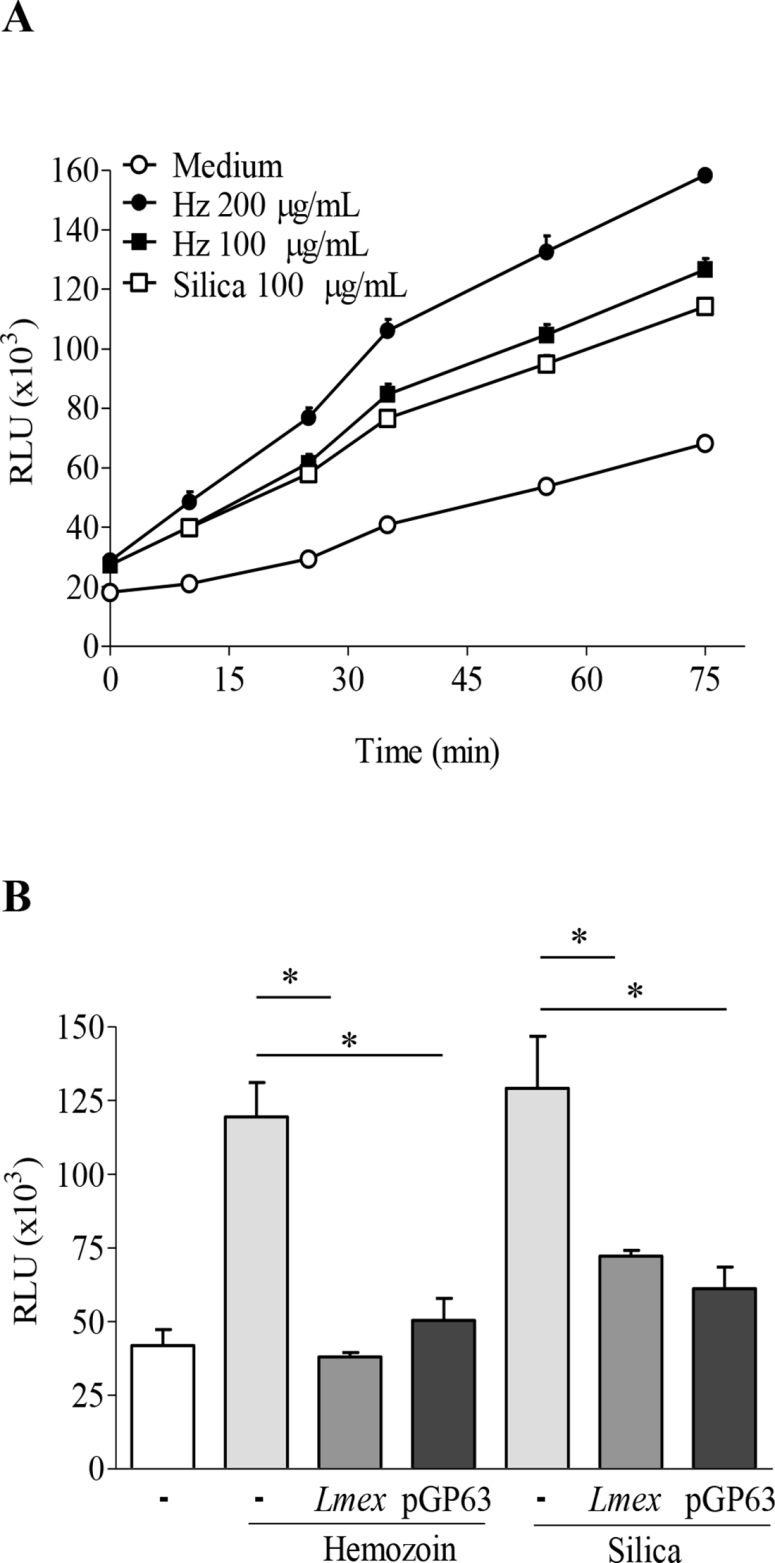
*Leishmania* parasites and purified GP63 inhibit generation of ROS induced by inflammasome activator. (**A** and **B**) PMA-differentiated THP-1 cells (0.1x10^6^ cells/100 μL) were stimulated with indicated concentration of HZ or silica (**A**) or pre-infected/incubated with either *Leishmania* or purified GP63 (pGP63) for 2 hrs, and then stimulated with 200 μg/mL of HZ or 100 μg/mL silica (**B**). After indicated time of incubation (**A**) or 1.5 hrs (**B**), ROS production was measured by fluorescence using DCFA. Differences were considered significant for p < 0.05. Data shows mean values with SEM of 3 independent experiments. (#) denotes significant changes between uninfected and HZ treated samples. (*) denotes significant changes of ROS production due to *Leishmania* infection or pGP63. Both *Leishmania* and purified GP63 inhibit ROS production induced by inflammasome activators.

Activation of the NLRP3 inflammasome due to DAMPs is often associated with ROS production and ROS-induced or -dependent signaling [38,39]. In this context the molecular basis of ROS generation has been under debate for some time and recent hypothesis include damage to mitochondria as a possible ROS-source and propose thioredoxin-interacting protein TXNIP may act as a ROS-sensor [40]. *Leishmania* has been shown to interfere with the generation of ROS and other microbicidal molecules [15] and has been described to be involved in the inflammasome activation [15]. Using known danger molecules like HZ and silica we determined, that both crystalline agents readily induce the generation of ROS in THP1 cells (Fig 3A). Therefore, we hypothesized that a *Leishmania*-dependent decrease of ROS-species or an impaired ROS production could be the basis of the diminished IL-1β maturation/release previously observed. Consequently, infection of THP-1 cells with *L*. *mexicana* led to an abrogated ROS production even after HZ or silica stimulation, supporting our hypothesis (Fig 3B). As our previous results suggested the possibility of a GP63-mediated inflammasome suppression, we included purified *L*. *mexicana* GP63 (pGP63) in our experimental setup. Pretreatment of THP cells with pGP63 from *L*. *mexicana* supernatant was also sufficient to reduce ROS levels to a similar extend as *Leishmania* infections (Fig 3B).

**Fig 4:**
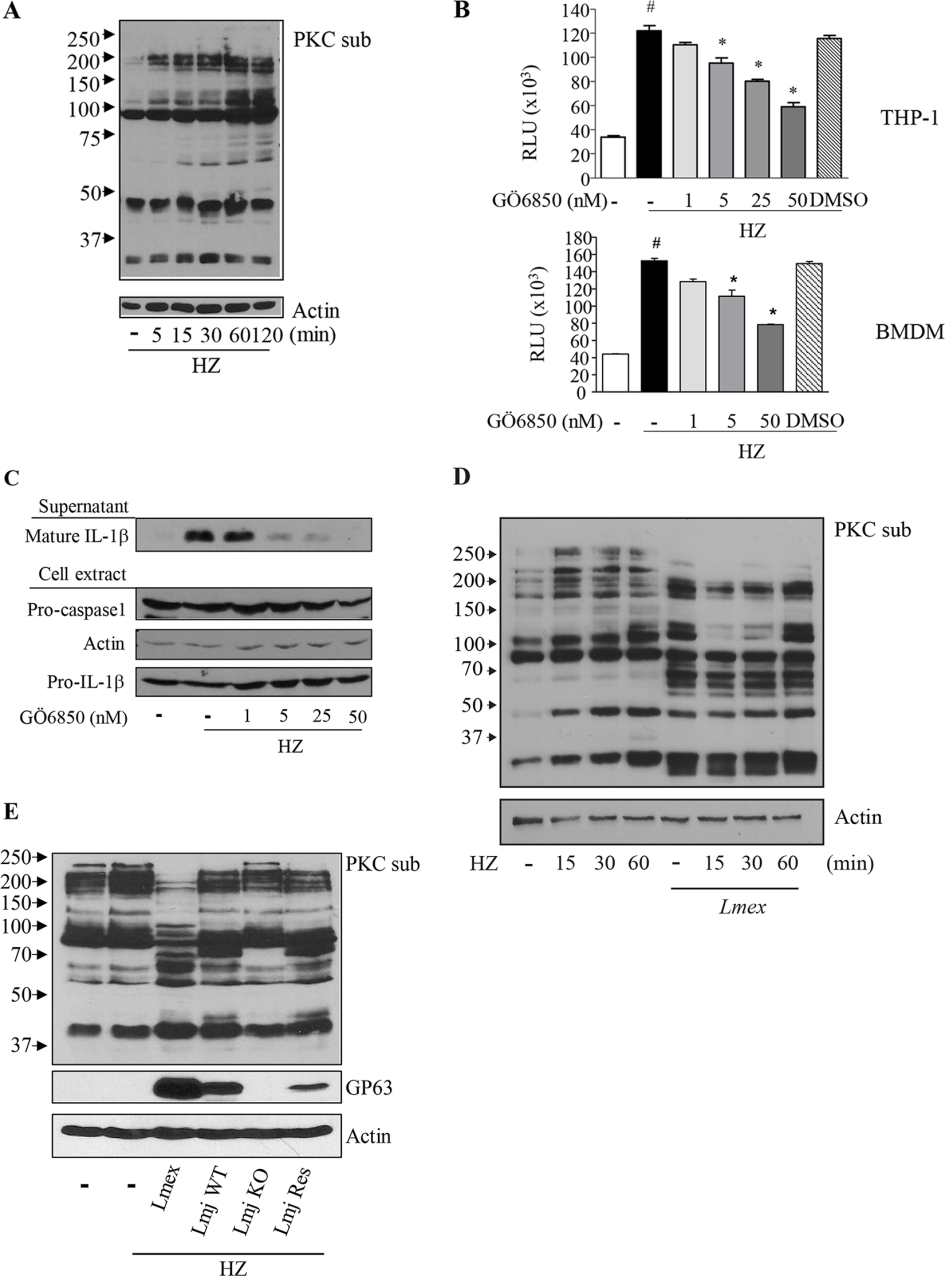
HZ-induced ROS and IL-1β production is mediated by PKC, which is in turn target of *Leishmania* parasites induced dephosphorylation. (**A**) PMA-differentiated THP-1 cells (1x10^6^ cells/mL) were stimulated with 200 μg/mL of HZ for indicated time points. (**B**) THP-1 or LPS-primed BMDMs (0.1x10^6^ cells/100 μL) were pre-treated with indicated concentrations of PCK inhibitor GÖ6850 for 30 min. After 1.5 hrs, ROS production was measured by fluorescence using DCFA. Data shows mean values with SEM of 3 independent experiments. Differences were considered significant for p < 0.05. (#) denotes significant changes between uninfected and HZ treated samples. (*) denotes significant changes of ROS production due to PKC inhibition Cells were pre-infected with the indicated *Leishmania* species for 2 hrs (**D**, **E**) or pre-treated with the indicated amounts of the PCK inhibitor GÖ6850 for 30 min (**C**) and then stimulated or not with 200 μg/mL of HZ for 6 hrs (**C**) or as indicated (**D**). Cell extracts of PMA-differentiated THP-1 were pre-infected with *L*. *major* GP63 wild type (*Lmj WT*) or *L*. *major* GP63^-/-^ (*Lmj KO*) parasites for 2 hrs and then stimulated or not with 200 μg/mL of HZ for 1 hr (**E**) Cell extracts were collected and subjected to Western blot analysis with the indicated antibodies (**A**, **C**, **D** and **E**). (-) indicates untreated samples. (PKCsub) denotes the usage of an antibody specific for PKC-dependent phosphorylation.

We previously presented evidence, that HZ-induced NLRP3 inflammasome activation is dependent on Syk activation and signaling [20]. Furthermore, in a variety of studies it has been suggested that Syk activation in turn can be coupled to PKC signaling [41]. PKC activation has previously been associated with ROS production [42,43]. Therefore, we sought to analyze, whether HZ affects PKC activation as well as PKC-mediated phosphorylation and if PKC-dependent signaling may be of importance for the HZ-driven ROS production and inflammasome activation. The analysis of PKC-dependent protein phosphorylation in PMA-differentiated THP-1 cells and BMDMs revealed, that HZ indeed led to an increased phosphorylation of PKC substrates (Fig 4A and S3 Fig). Specific inhibition of PKC using the PKC-inhibitor GÖ6850 [44] was able to counteract the augmented PKC-substrate phosphorylation levels after application of HZ (S4 Fig). The examination of ROS production after the loss of PKC-dependent phosphorylation and PKC-signaling revealed a significant decrease of intracellular ROS-generation in both THP-1 cells (Fig 4B, upper panel) and LPS-primed BMDM (Fig 4B, lower panel). Additionally, to analyze whether PKC-mediated ROS generation was connected to the attenuated IL-1β maturation and/or release we investigated IL-1β levels after PKC-inhibition. In accordance with the data shown previously, IL-1β maturation is abrogated after suppression of PKC-dependent signaling through the application of the PKC inhibitor GÖ6850 (Fig 4C). Interestingly, we already established in the past that PKC activation can be negatively modulated by *Leishmania* infections [45]. Consequently, experiments with *L*. *mexicana* preceding HZ stimulation showed that PKC-dependent phosphorylation in this context is clearly altered by *Leishmania* parasites presenting a possible explanation for our previous observations (Fig 4D). Through the use of *L*. *major* GP63 wild type (*Lmj WT*) and *L*. *major* GP63^-/-^ (*Lmj KO*) parasites we were able to further substantiate the dependency of PKC-dependent ROS-reduction on GP63 activity in both THP-1 and BMDM cells (Fig 4E) Taken together our results suggest that PKC activation is a signaling event upstream of IL-1β production after HZ stimulation, which is disrupted by *Leishmania*. In conclusion the analysis of ROS-production and PKC-signaling after infection, the use of pGP63, GP63^-/-^ parasites and the chemical inhibition of PKC, suggest that PKC-dysregulation most likely through GP63 impairs IL-1β release.

### Leishmanial GP63 cleaves components of the NLRP3 inflammasome complex

**Fig 5:**
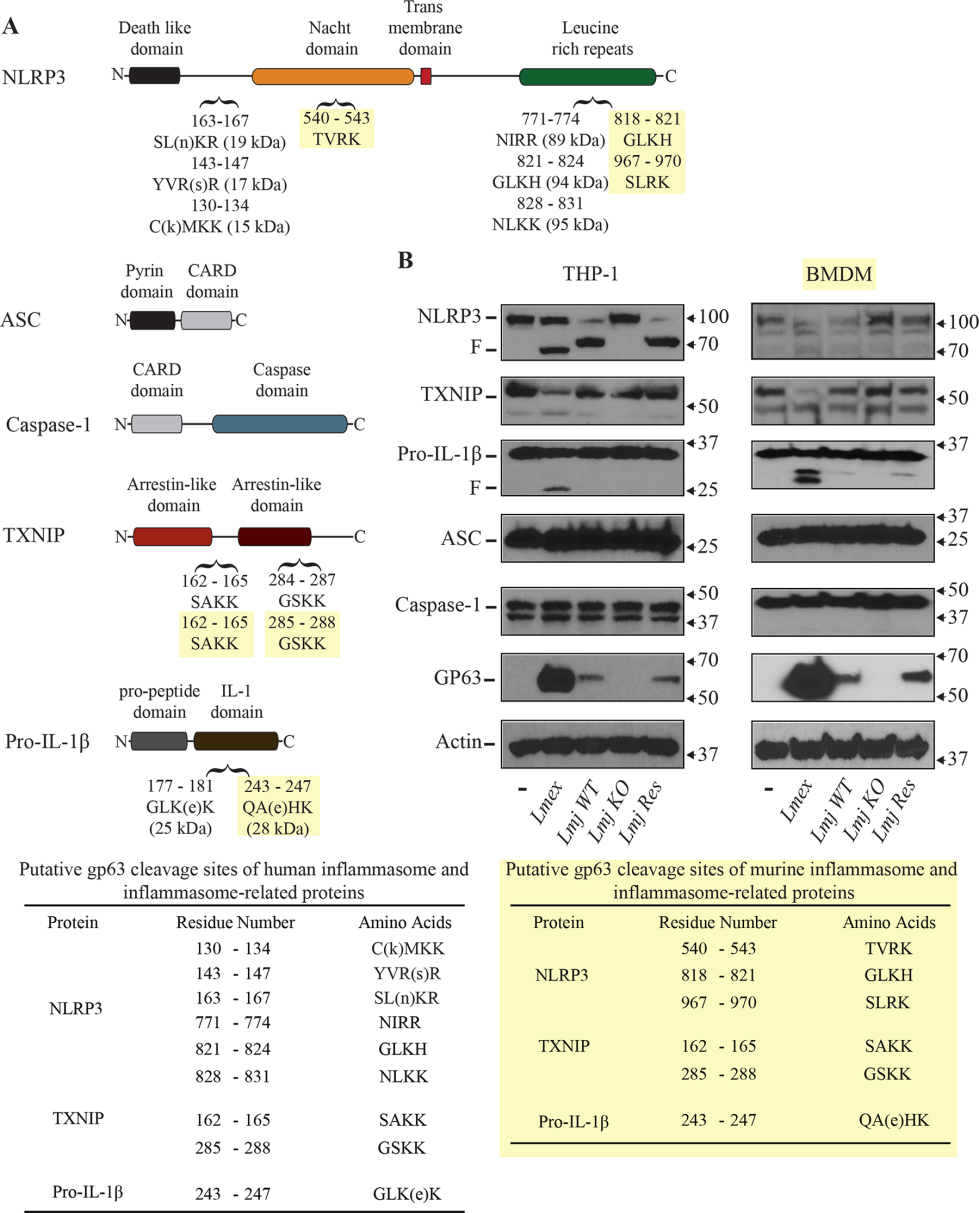
Analysis of inflammasome-related and inflammasome-complex proteins for GP63-induced manipulation. (**A**) In silico GP63 cleavage sites analysis of indicated components of the NLRP3 inflammasome complex was done using the ScanProsite platform. (**B**) PMA-differentiated THP-1 cells or LPS-primed BMDMs were infected with *Leishmania mexicana* (*Lmex*), *L*. *major* GP63 wild type (*Lmj WT*), *L*. *major* GP63^-/-^ (*Lmj KO*) or *L*. *major* GP63 rescue (*Lmj Res*) for 2 hrs. Cells were washed, cell extracts were collected and subjected to Western blot analysis with the indicated antibodies. (F) denotes the generation of GP63-dependent cleavage fragments. (**C**) Summary of potential cleavage sites in murine and human inflammasome and inflammasome-related proteins. Data shown is representative of three independent experiments.

Our previous data showed that *Leishmania* infections of macrophages prevent the maturation of pro-IL-1β to mature IL-1β upon stimulation possibly intervening with a host protective effect. Thus far, the impairment of IL-1β after infection was attributed to a suppression of PKC-dependent signaling and the loss of ROS production. As the enclosed data supports that GP63 is closely associated with these events, we wanted to examine if *Leishmania* parasites and GP63 may also interfere with inflammasome activation through proteolytic cleavage of inflammasome components. We and others demonstrated that GP63 can cleave targets containing the following amino acid-motives: polar/hydrophobic/basic/basic amino acids (P_1_- P’_1_-P’_2_-P’_3_) [43,46]. A first indication for GP63-dependent cleavage of inflammasome components was obtained by experiments using BMDMs. There, we observed that after infection with *Leishmania* we were able to observe cleavage of pro-IL-1β (Fig 1C). An *in silico* sequence analysis for putative GP63 cleavage sites revealed the possibility of additional GP63 cleavage sites in the sequences of inflammasome complex and associated proteins. Thus, the sequences of human and murine NLRP3; pro-IL1β and TXNIP—a protein that has been suggested to possibly be involved in ROS-mediated inflammasome activation [40]–contain putative cleavage sites for GP63 (Fig 5A and 5C). As GP63 facilitated cleavage is not necessarily restricted to the proposed cleavage motif and to confirm our *in silico* findings we performed Western blotting analysis of infected THP1 and LPS-primed BMDM cells. In accordance with the *in silico* data, GP63 seemed to be able to directly interfere with the inflammasome complex. Thus, we observed cleavage of NLRP3 after infection with *Leishmania* (Fig 5). This process was GP63-dependent as illustrated by the results for *L*. *major* wt, GP63KO and GP63 rescue parasites. Although, the *in silico* analysis did not suggest a GP63-mediated cleavage of ASC or Caspase-1 we choose to analyze both as they are an integral part of the inflammasome complex. Neither pro-caspase-1 nor ASC showed any cleavage after infection. In addition, as anticipated cleavage and/or cleavage fragments were detected for pro-IL-1β and TXNIP in lysates of cells infected with *L*. *mexicana*, *L*. *major wt* or *L*. *major* GP63 rescue expressing GP63, but not in cells infected with *L*. *major* GP63 KO. Taken together our data suggests that *Leishmania* is able to impair inflammasome activation through different GP63-dependent alterations of proteins and signaling pathways.

### *Leishmania*-mediated suppression of ROS-independent IL-1β production

**Fig 6:**
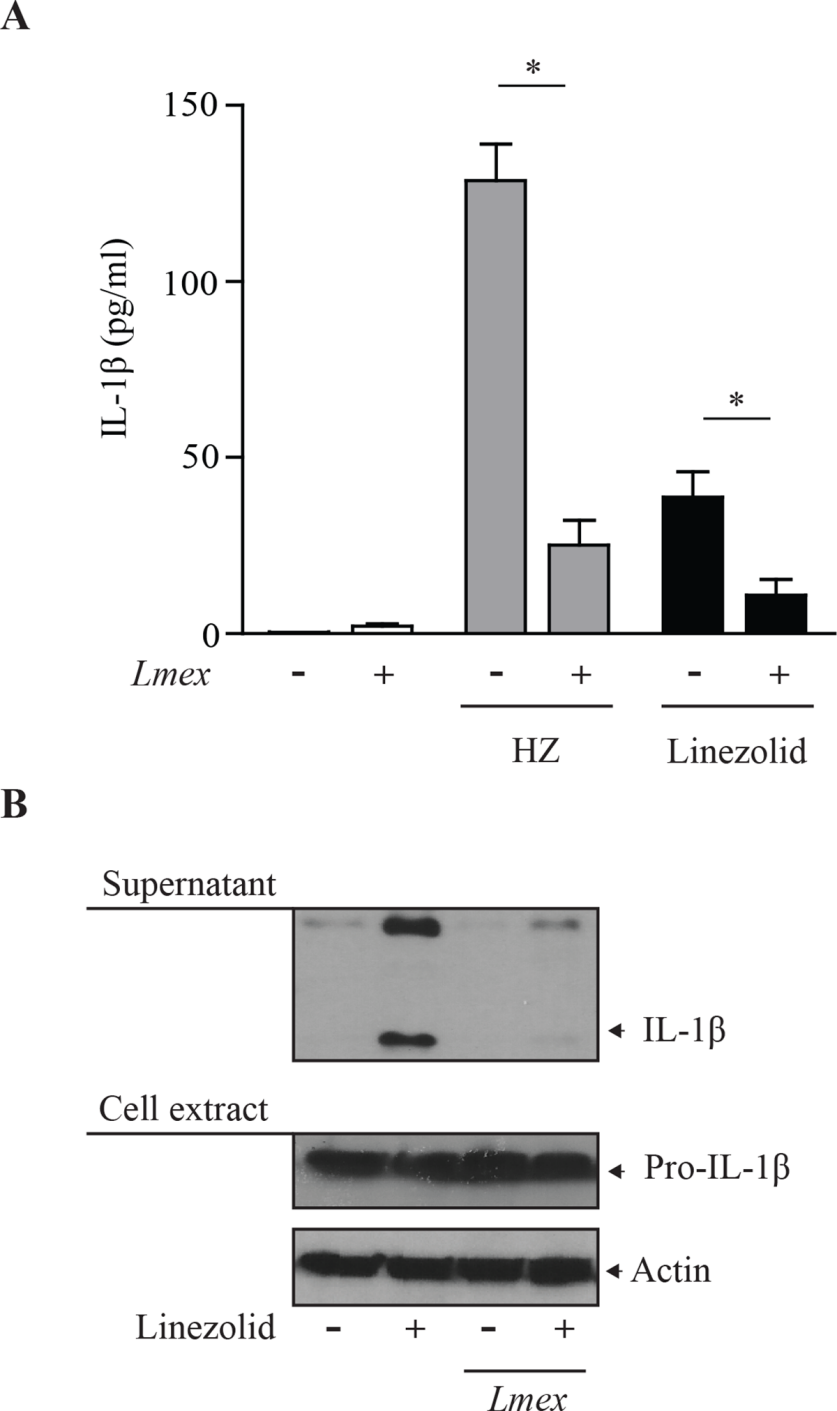
ROS-independent IL-1β generation is dampened after infection with Leishmania. (**A**) LPS-primed BMDMs were infected with *Leishmania mexicana* (*Lmex*), for 2 hrs. Cells were washed three times and incubated in serum free MEM alpha medium for 16 hrs in the presence of linezolid (100 μg/ml) or HZ (200 μg/ml) as indicated. Supernatants were collected and subjected to IL-1β specific ELISA analysis. Data shows mean values with SEM of 3 independent experiments. Differences were considered significant for p < 0.05(*). (**B**) PMA-differentiated THP-1 cells were infected with *Leishmania mexicana* (*Lmex*). Cells were washed three times and incubated in serum free MEM alpha medium for 16 hrs in the presence of linezolid (100 μg/ml) as indicated. Afterwards, cell extracts were collected and subjected to Western blot analysis with the indicated antibodies. Data shown is representative of three independent experiments.

A controversial question of inflammasome activation is the dependency of ROS for the activation and assembly of the complex. Recent data associates mitochondrial damage with the activation of the NLRP3 inflammasome. In this context cardiolipin seems to work as a DAMP, able to induce inflammasome complex formation and ultimately IL-1β. The previous data presented different ways how leishmanial GP63 can suppress inflammasome activity. This included the cleavage of NLRP3. Therefore, we wanted to know whether these processes might also have a direct effect on IL-1β production. Thus, we observed the IL-1β generation in a ROS-independent experimental setup using the antibiotic linezolid [19]. Linezolid stimulation of either THP-1 or LPS-primed BMDM cells resulted in the maturation of IL-1β as previously published (Fig 6A and 6B). When cells were infected prior to linezolid stimulation, IL-1β release appeared reduced for both murine and human cells. In accordance to previously shown data Leishmania infection alone did only lead to a minimal production of mature IL-1β (Fig 6A). Taken together this finding may indicate, that the infection of cells with *Leishmania* can abrogate both ROS-dependent and -independent inflammasome activation, possibly through different mechanisms as both ROS-inducing signaling events are blocked and inflammasome components are cleaved due to the leishmanial protease GP63.

## Discussion

*Leishmania* parasites have evolved many mechanisms to hijack macrophage microbicidal functions in order to survive and proliferate within the phagocytes. In the present work we addressed how *Leishmania* parasites can attenuate IL-1β production through the leishmanial virulence factor GP63 during infection. In the past, the activity of inflammasomes and the associated production of especially IL-1β has been correlated with the host protection against parasitic infections, for instance in the case of T. *cruzi* or *T*. *gondii* [47,48,49]. In the case of *Leishmania* parasites the importance and the role of inflammasomes and IL-1β is very controversially discussed, mainly due to the use of different *Leishmania* species, different leishmanial developmental stages and different infection models. Previous work indicated the possibility of a species-dependent dysregulation of inflammasomes and inflammasome-related pathways and implicated different leishmanial virulence factors. Reports showed that *L*. *donovani* and *L*. *tropica* do not induce IL-1β production, and negatively modulate the capacity of IFN-primed human or LPS-primed murine peritoneal macrophages to produce IL-1β upon activation [10,50,51]. The focus of several studies was a parasite-mediated dysregulation of IL-1β on a transcriptional level after infection of human [52,53] or murine phagocytes [54]. In this context, Hatzigeorgiou and collaborators [55] implicated a LPG-dependent interference with IL-1β mRNA that translated into both decreased stability and production of IL-1β mRNA and consequently reduced transcription of the IL-1β gene [52,55]. However, data of Cillari et al. [54] and Gurung et al. [56] indicated that *L*. *major* infections might increase inflammasome activity and cytokine production during long term infections. Some reports indicate that the role the inflammasome is dependent on the model for infection with a possible delay in the resolution of cutaneous lesions in the absence of IL-1β [33,34]. Thus indications exist for a possible inflammasome-mediated host protective mechanism in some murine models of infection. In contradictory reports working with the new world *Leishmania* species *L*. *amazoniensis*, parasites have been described to be able to both suppress and induce IL-1β secretion by infected cells. On the one hand a study by Ji et al. provided data that the infection of C57Bl/6 mice with *L*. *amazoniensis* led to a delay in the secretion of chemokines and cytokines including IL-1β *in vivo* [11]. On the other hand a recent report of Lima-Junior et al. presented data that inflammasomes and IL-1β are involved in the control of *L*. *amazoniensis* infections of C57Bl/6 mice as shown by *in vitro* and *in vivo* studies using mice and BMDMs of deficient in IL-1β production (including caspase-1 and NLRP3 KO mice) [13].

Our infection experiments with *L*. *major* and *L*. *mexicana* revealed an attenuated capability of macrophages to produce and secrete IL-1β when stimulated with the specific and well characterized NLRP3 inflammasome agonist HZ [20]. This we observed in C57Bl/6-derived BMDMs as well as after infection of human cells, which may indicate a role in the circumvention of a host protective mechanism by *Leishmania*. In PMA-differentiated THP-1 cells as well as in LPS-primed macrophages, *L*. *major* and *L*. *mexicana* inhibited NLRP3 inflammasome activation as indicated by reduced levels of secreted IL-1β. A possible explanation to the divergent result to previous reports, like Lima-Junior et al. [13], could be the difference in *Leishmania* spp. used. As introduced before, especially for *L*. *amazoniensis* previous data has been controversial. Furthermore, it is to be noted, that *L*. *amazoniensis* exhibits a rather unique pathogenesis and a very peculiar intracellular compartmentalization after host infection, which is not observable with the species used in our report [57]. Another crucial difference is potentially the experimental setup, specifically the time of incubation used to detect IL-1β production. It is in fact conceivable that longer periods of infection as examined in the work of Lima-Junior et al. may affect the secretion of IL-1β through cell death related events [58,59]. On this note, data published by Gomes et al. is noteworthy [60]. In their experiments using *L*. *braziliensis* IL-1β production was dependent on the developmental form of the parasite used. Infections with amastigotes led to IL-1β maturation while promastigotes did not. Thus the transition from promastigotes to amastigotes during infection may be of the essence for a *Leishmania-*mediated effect on IL-1β maturation. In addition, we want to point out the fact that infection in our experiments preceded inflammasome activation while previous work predominantly analyzed IL-1β levels over time after infection *in vivo* or after stimulation of cells with an inflammasome inducing agent like LPS and subsequent infection *in vitro* [11,13]. Collectively, our results may indicate that an initial block of IL-1 maturation may prevent a host protective effect, thus facilitating parasite survival.

Importantly, none of the previous reports analyzed the possibility of a GP63-mediated effect on IL-1β maturation. In our study, we were able to observe an attenuated IL-1β production using not only parasites but *Leishmania* culture supernatants, leishmanial secretome and exosome preparations as well, all of which have been shown to contain the metalloprotease GP63 [2,28]. Moreover, purified GP63 exhibited similar effects when used on human THP-1 cells prior to inflammasome activation with HZ. We previously showed that the malaria pigment HZ elevates ROS-levels leading to inflammasome activation [20]. ROS have been shown to contribute to parasite clearance and are inhibited by *Leishmania* parasites [1]—an effect that can be mediated through the activity of the metalloprotease GP63. Although at this point we cannot rule out the involvement of other leishmanial factors in the inhibition of ROS generation, our data and the usage of purified recombinant GP63 strongly suggests an important role of the protease in this context. Our work identified PKC-signaling as the mechanism upstream of the observed ROS induction after treatment of THP-1 cells with HZ. In the past, different studies presented evidence for a role of PKC both upstream and downstream of ROS generation [42,43,61]. Our results show that PKC-signaling and especially PKC-dependent ROS-generation can mediate inflammasome activation. Some studies previously indicated similar implications for PKC in the activation of the NLRC4 inflammasome [62]. In this context PKCδ-mediated NLRC4-phosphorylation was suggested as the basis of the observed effects [62]. Thus, we here clarify how PKC signaling may also affect NLRP3 inflammasome activation in response to DAMPs like HZ.

In agreement with previous data we were also able to establish that *Leishmania* is capable to alter the previously introduced HZ-mediated PKC activation after *Leishmania* infection. As a consequence stimulated cells exhibited a loss of IL-1β production. Initial reports using *L*. *donovani* suggested that LPG was involved in the alteration of PKC-signaling as purified LPG prevented PKC activation in macrophages after stimulation with either LPS or PKC-activators [63]. Nevertheless, it has been described in recent years that the inhibition of the oxidative burst in macrophages as well as the associated signaling events, including PKC, were in part mediated by the parasites surface molecules LPG and GP63 [45,64] after infection. Our findings are also corroborated by previous reports that revealed a GP63-mediated interference with PKC-signaling and PKC targets. In this regard, Corradin et al. presented evidence that the PKC substrates myristoylated alanine-rich C kinase substrate (MARCKS)-related protein (MacMARCKS) and myristoylated alanine-rich C kinase substrate (MARCKS), the latter which is of importance for cell motility, adhesion, endo-, exo- and phagocytosis as well as for the interplay of calmodulin and PKC signaling, [65] are targeted by GP63 for cleavage [66,67].

The interference of pathogens with inflammasomes has been shown in a number of bacterial or viral infections. This includes the expression of decoy proteins that bind NLRs or ASC, factors that block caspase-1 activity or scavenger receptors for IL-1β [68]. Interestingly, this also includes Zmp1 a Zn^2+^-metalloprotease expressed by *Mycobacteria spp*. that interferes with caspase-1 activation [69]. The leishmanial Zn^2+^-metalloprotease GP63 has been shown to facilitate cleavage of a multitude of cellular substrates, most notably cellular phosphatases. *In silico* data using the GP63-cleavage motif [46] indicated a possible GP63-mediated processing of inflammasome or inflammasome-associated proteins, including NLRP3 and pro-IL-1β, which we were able to confirm in infection experiments of both murine BMDMs and human THP-1 macrophages. Interestingly, we observed that one of the GP63-cleaved proteins was TXNIP, which has been shown to facilitate ROS-dependent inflammasome activation [40]. Thus, our data indicates that *Leishmania* may employ different GP63-linked strategies to impair secretion and maturation of IL-1β during infection, the downregulation of ROS on the one hand and the cleavage of inflammasome and inflammasome-related proteins on the other hand. The relevance of the latter mode of inflammasome inhibition is illustrated by the diminished release of IL-1β after stimulation of cells with linezolid, a ROS-independent inflammasome inducer [19].

IL-1β has been associated with the control of parasitic infections possibly including various *Leishmania* species. Collectively, we here provide evidence that *Leishmania major* and *mexicana* parasites are able to dampen IL-1β secretion during initial stages of infection, rendering cells non-responsive towards stimulation of the NLRP3 inflammasome. This may substantiate a host protective mechanism that has been suggested previously [33]. Moreover, we here show that the observed reduction of IL-1β maturation after infection takes place in both a murine and a human infection model. Our finding that the parasites can impair cytokine secretion through both the downregulation of ROS and possibly the proteolytic cleavage of inflammasome and inflammasome-related proteins strongly supports an important role of this mechanism in the formation of infection. Thus, our data presents a novel way whereby *Leishmania* ensures the infection of their target cells emphasizing the parasites ability to overcome host protective functions during infection.

## Supporting Information

S1 Fig Activation of the NLRP3 inflammasome by HZ and other known agonists. PMA-differentiated THP-1 cells (1x10^6^ cells/mL) were stimulated with indicated amounts of different agonists of the NLRP3 inflammasome including HZ, silica, asbestos and MSU for 6 hrs. Supernatant and cell extracts were collected and subjected to Western blot analysis with the indicated antibodies.
(TIF)

S2 Fig *Leishmania mexicana* infection alone does not cause IL-1β secretion. PMA-differentiated THP-1 cells were infected with *Leishmania mexicana* (*Lmex*). Cells were washed three times and incubated in serum free MEM alpha medium for 6 hrs in the presence of HZ (200 μg/ml) as indicated. Afterwards cell extracts were collected and subjected to Western blot analysis with the specified antibodies indicated. Data shown is representative of three independent experiments.
(TIF)

S3 Fig PKC-stimulation in BMDMs by hemozoin. LPS-primed BMDMs were stimulated with 200 μg/mL of HZ for indicated time points. Afterwards cell extracts were collected and subjected to Western blot analysis with the specified antibodies indicated. Data shown is representative of two independent experiments. (PKCsub) denotes the usage of an antibody specific for PKC-dependent phosphorylation.
(TIF)

S4 Fig Inhibition of PKC-signaling by GÖ6850. PMA-differentiated THP-1 or LPS-primed BMDMs were pretreated for 30 min with the indicated amounts of the PKC-inhibitor GÖ6850 and stimulated with 200 μg/mL of HZ for 30min. Afterwards, cell extracts were collected and subjected to Western blot analysis with the specified antibodies indicated. Data shown is representative of two independent experiments. (PKCsub) denotes the usage of an antibody specific for PKC-dependent phosphorylation
(TIF)
