# Supplementary figures and images for: PKC/ROS-Mediated NLRP3 Inflammasome Activation Is Attenuated by Leishmania Zinc-Metalloprotease during Infection
Source: PLoS Negl Trop Dis. 2015 Jun 26;9(6):e0003868. doi: 10.1371/journal.pntd.0003868 (PMC4482689; doi:10.1371/journal.pntd.0003868)

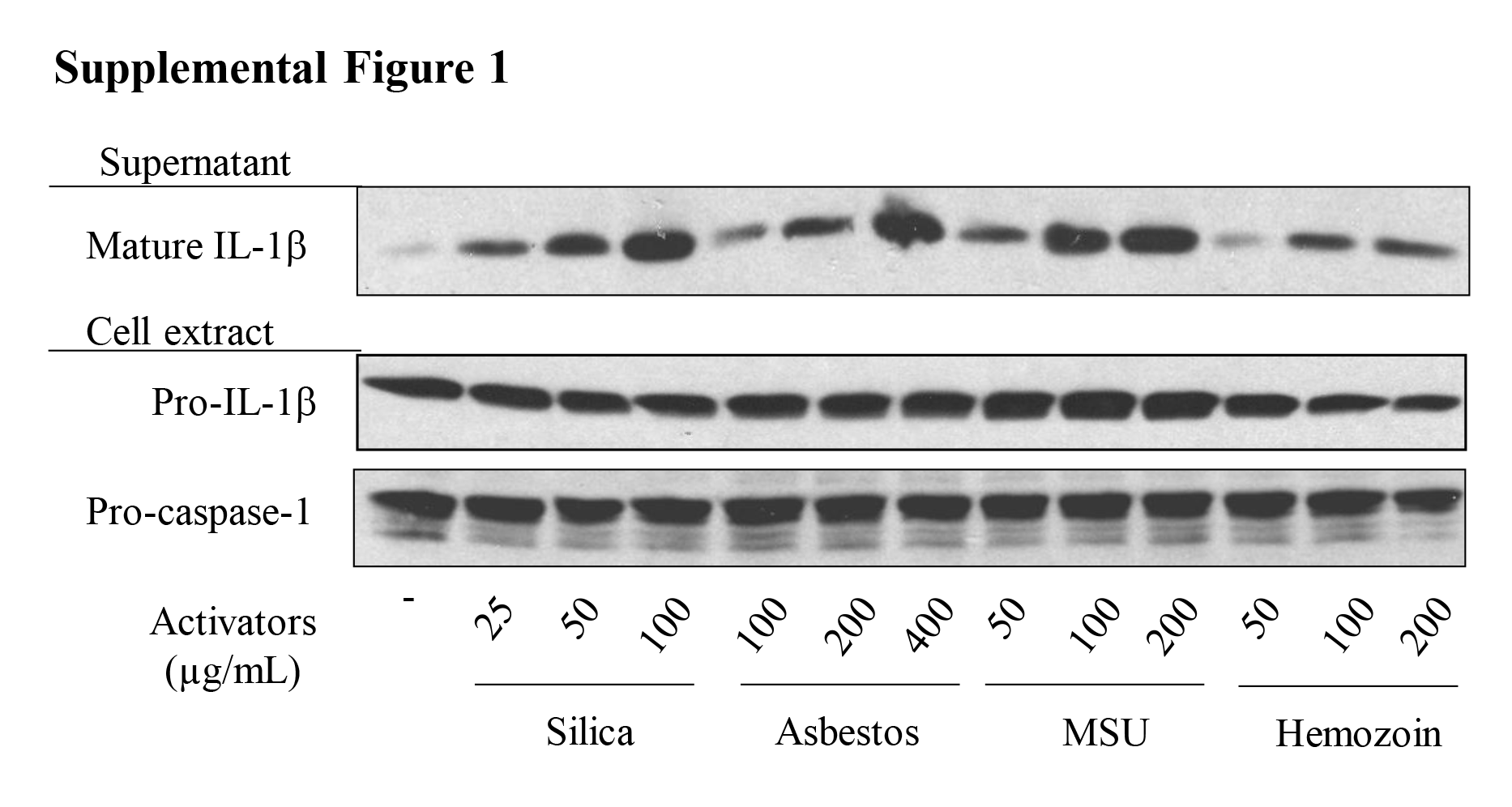

Supplement: S1 Fig — PMA-differentiated THP-1 cells (1x10^6^ cells/mL) were stimulated with indicated amounts of different agonists of the NLRP3 inflammasome including HZ, silica, asbestos and MSU for 6 hrs. Supernatant and cell extracts were collected and subjected to Western blot analysis with the indicated antibodies. (TIF) [file pntd.0003868.s001.tif]

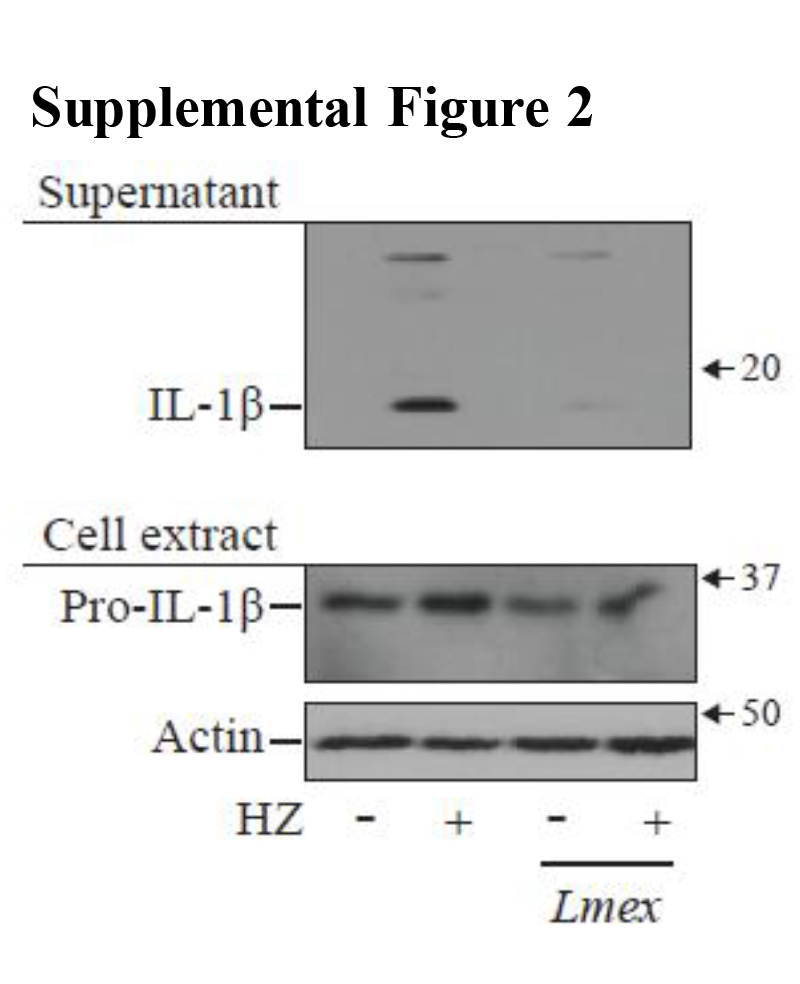

Supplement: S2 Fig — PMA-differentiated THP-1 cells were infected with Leishmania mexicana (Lmex). Cells were washed three times and incubated in serum free MEM alpha medium for 6 hrs in the presence of HZ (200 μg/ml) as indicated. Afterwards cell extracts were collected and subjected to Western blot analysis with the specified antibodies indicated. Data shown is representative of three independent experiments. (TIF) [file pntd.0003868.s002.tif]

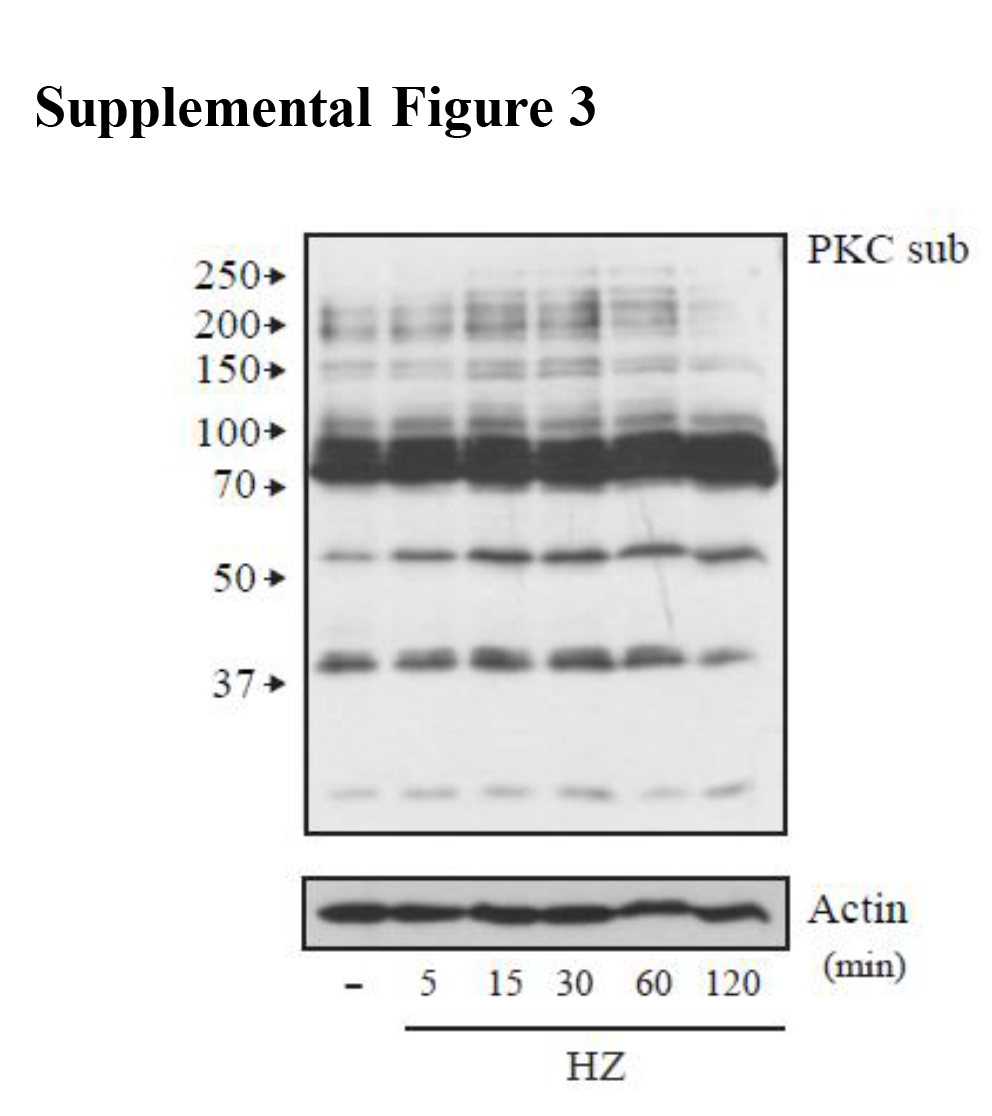

Supplement: S3 Fig — LPS-primed BMDMs were stimulated with 200 μg/mL of HZ for indicated time points. Afterwards cell extracts were collected and subjected to Western blot analysis with the specified antibodies indicated. Data shown is representative of two independent experiments. (PKCsub) denotes the usage of an antibody specific for PKC-dependent phosphorylation. (TIF) [file pntd.0003868.s003.tif]

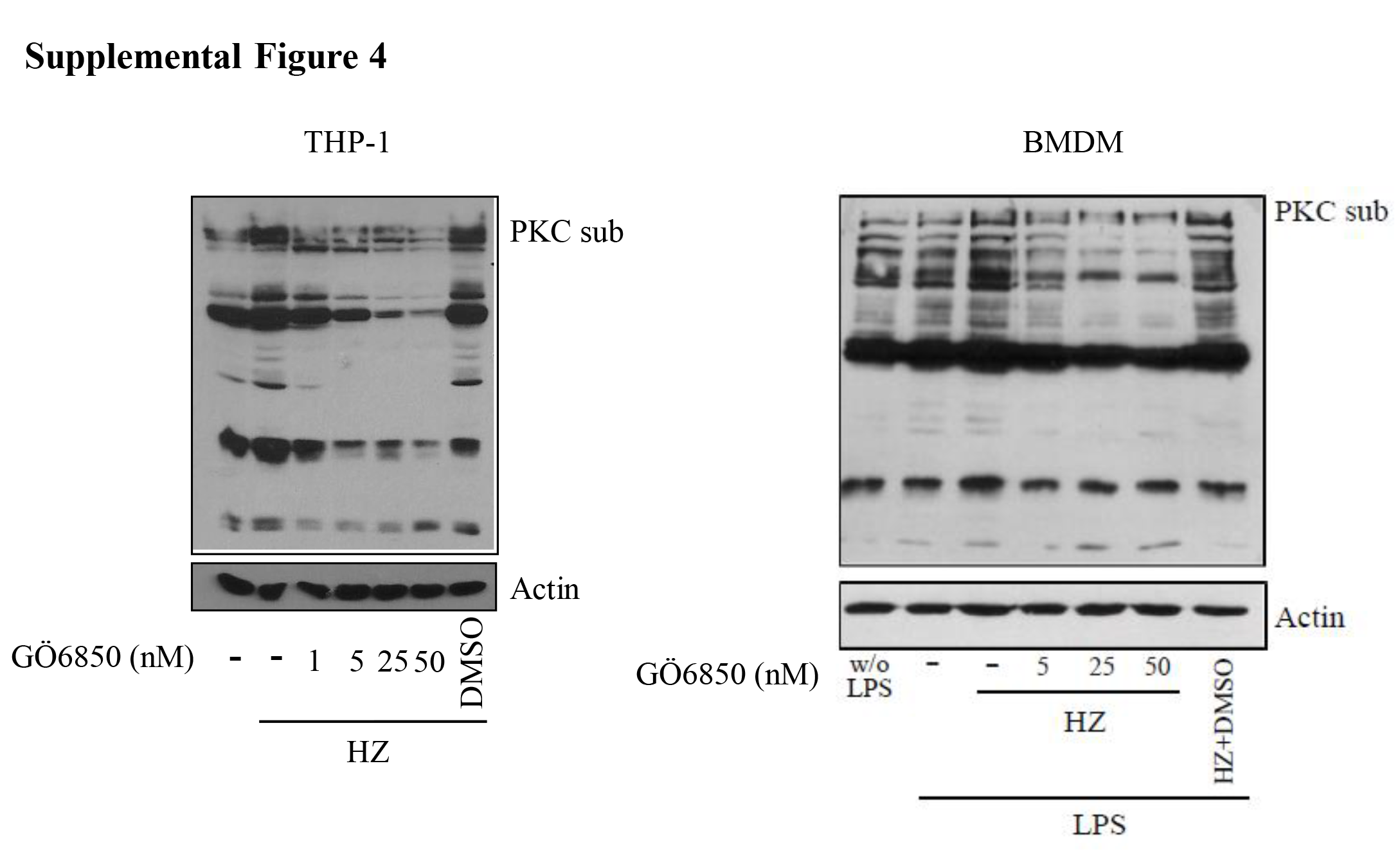

Supplement: S4 Fig — PMA-differentiated THP-1 or LPS-primed BMDMs were pretreated for 30 min with the indicated amounts of the PKC-inhibitor GÖ6850 and stimulated with 200 μg/mL of HZ for 30min. Afterwards, cell extracts were collected and subjected to Western blot analysis with the specified antibodies indicated. Data shown is representative of two independent experiments. (PKCsub) denotes the usage of an antibody specific for PKC-dependent phosphorylation (TIF) [file pntd.0003868.s004.tif]
